# Is there a preferred platinum and fluoropyrimidine regimen for advanced HER2-negative esophagogastric adenocarcinoma? Insights from 1293 patients in AGAMENON–SEOM registry

**DOI:** 10.1007/s12094-024-03388-6

**Published:** 2024-02-15

**Authors:** Aranzazu Arias-Martinez, Eva Martínez de Castro, Javier Gallego, Virginia Arrazubi, Ana Custodio, Ana Fernández Montes, Marc Diez, Raquel Hernandez, María Luisa Limón, Juana María Cano, Rosario Vidal-Tocino, Ismael Macias, Laura Visa, Marta Martin Richard, Tamara Sauri, Cinta Hierro, Mireia Gil, Paula Cerda, Elia Martínez Moreno, Nieves Martínez Lago, Antonio José Mérida-García, Lucía Gómez González, Francisco Javier García Navalón, Maribel Ruiz Martín, Gema Marín, Flora López-López, Ana Belen Ruperez Blanco, Alejandro Francisco Fernández, Paula Jimenez-Fonseca, Alberto Carmona-Bayonas, Felipe Alvarez-Manceñido

**Affiliations:** 1 Doctoral Program in Pharmacy, Universidad de Granada, Barrio Verxeles n°13 2°, CP 27850 Granada, Viveiro Spain; 2 Medical Oncology Department, Hospital Universitario Marqués de Valdecilla, IDIVAL, Santander, Spain; 3 Medical Oncology Department, Hospital General Universitario de Elche, Elche, Spain; 4 Medical Oncology Department, Hospital Universitario de Navarra, IdiSNA, Pamplona, Spain; 5 Medical Oncology Department, Hospital Universitario La Paz, CIBERONC, CB16/12/00398, Madrid, Spain; 6 Medical Oncology Department, Complejo Hospitalario Universitario de Orense, Orense, Spain; 7 Medical Oncology Department, Hospital Universitario Vall d’Hebron, VHIO, Barcelona, Spain; 8 Medical Oncology Department, Hospital Universitario de Canarias, Tenerife, Spain; 9 Medical Oncology Department, Hospital Universitario Virgen del Rocío, Seville, Spain; 10 Medical Oncology Department, Hospital General Universitario de Ciudad Real, Ciudad Real, Spain; 11 Medical Oncology Department, Complejo Asistencial Universitario de Salamanca - IBSAL, Salamanca, Spain; 12 Medical Oncology Department, Hospital Universitario Parc Tauli, Sabadell, Spain; 13 Medical Oncology Department, Hospital Universitario El Mar, Barcelona, Spain; 14 Medical Oncology Department, Instituto Catalán de Oncología (ICO), L’Hospitalet de Llobregat, Barcelona, Spain; 15 Medical Oncology Department, Hospital Clinic, Barcelona, Spain; 16 Medical Oncology Department, Instituto Catalán de Oncología (ICO)-Badalona, Barcelona; Badalona-Applied Research Group in Oncology (B-ARGO), Badalona, Spain; 17 Medical Oncology Department, Hospital General Universitario de Valencia–Ciberonc CB16/12/0035, Valencia, Spain; 18 Medical Oncology Department, Hospital Universitario Santa Creu y Sant Pau, Barcelona, Spain; 19 Medical Oncology Department, Hospital Universitario de Fuenlabrada, Madrid, Spain; 20 Medical Oncology Department, Complejo Hospitalario Universitario de Ferrol, Ferrol, Spain; 21 Medical Oncology Department, Complejo Asistencial de Zamora, Zamora, Spain; 22 Medical Oncology Department, Hospital General Universitario de Alicante, Alicante, Spain; 23 Medical Oncology Department, Hospital Universitario de Son Llatzer, Mallorca, Spain; 24 Medical Oncology Department, Complejo Asistencial Universitario de Palencia, Palencia, Spain; 25 Medical Oncology Department, Hospital Universitario Virgen de la Arrixaca, Murcia, Spain; 26 Medical Oncology Department, Hospital Universitario del Sureste, Madrid, Spain; 27 Medical Oncology Department, Hospital Universitario de Toledo, Toledo, Spain; 28 Medical Oncology Department, Complejo Hospitalario Universitario de Pontevedra, Pontevedra, Spain; 29 Medical Oncology Department, Hospital Universitario Central de Asturias, ISPA, Oviedo, Spain; 30 Hematology and Medical Oncology Department, Hospital Universitario Morales Meseguer, University of Murcia, IMIB, Murcia, Spain; 31 Pharmacy Department, Hospital Universitario Central de Asturias, Oviedo, Spain

**Keywords:** Advanced esophagogastric cancer, Chemotherapy, Cisplatin, Fluoropyrimidine, Oxaliplatin, Survival

## Abstract

**Background:**

The optimal chemotherapy backbone for HER2-negative advanced esophagogastric cancer, either in combination with targeted therapies or as a comparator in clinical trials, is uncertain. The subtle yet crucial differences in platinum-based regimens' safety and synergy with combination treatments need consideration.

**Methods:**

We analyzed cases from the AGAMENON–SEOM Spanish registry of HER2-negative advanced esophagogastric adenocarcinoma treated with platinum and fluoropyrimidine from 2008 to 2021. This study focused exclusively on patients receiving one of the four regimens: FOLFOX (5-FU and oxaliplatin), CAPOX (capecitabine and oxaliplatin), CP (capecitabine and cisplatin) and FP (5-FU and cisplatin). The aim was to determine the most effective and tolerable platinum and fluoropyrimidine-based chemotherapy regimen and to identify any prognostic factors.

**Results:**

Among 1293 patients, 36% received either FOLFOX (*n* = 468) or CAPOX (*n* = 466), 20% CP (*n* = 252), and 8% FP (*n* = 107). FOLFOX significantly increased PFS (progression free survival) compared to CP, with a hazard ratio of 0.73 (95% CI 0.58–0.92, *p* = 0.009). The duration of treatment was similar across all groups. Survival outcomes among regimens were similar, but analysis revealed worse ECOG–PS (Eastern Cooperative Oncology Group–Performance Status), > 2 metastatic sites, bone metastases, hypoalbuminemia, higher NLR (neutrophil-to-lymphocyte ratio), and CP regimen as predictors of poor PFS. Fatigue was common in all treatments, with the highest incidence in FOLFOX (77%), followed by FP (72%), CAPOX (68%), and CP (60%). Other notable toxicities included neuropathy (FOLFOX 69%, CAPOX 62%), neutropenia (FOLFOX 52%, FP 55%), hand–foot syndrome in CP (46%), and thromboembolic events (FP 12%, CP 11%).

**Conclusions:**

FOLFOX shown better PFS than CP. Adverse effects varied: neuropathy was more common with oxaliplatin, while thromboembolism was more frequent with cisplatin.

**Supplementary Information:**

The online version contains supplementary material available at 10.1007/s12094-024-03388-6.

## Introduction

Gastric cancer ranks as the fifth most common neoplasm and the fourth leading cause of cancer-related deaths worldwide. Despite a reduction in incidence in recent years, along with advancements in diagnostic methods and surgical techniques, about 30% of patients with resected gastric cancer undergo relapse and subsequently require systemic treatment [1]. This is particularly concerning considering that at diagnosis, 50% of gastric cancer patients present with an unresectable disease—10% with locally advanced and 40% with metastatic stages [2].

Chemotherapy enhances overall survival (OS) in advanced disease by approximately 6–7 months compared to best supportive care, leading to median survival times of 9–11 months, even in patients with good functional status [3]. Now that chemotherapy combinations with new therapies, such as immunotherapy and anti-claudin agents, are emerging, it is worthwhile to briefly recall the complex historical development of various chemotherapy regimens to understand how we have arrived at the current state of treatment. In the 1990s, two-phase 3 randomized clinical trials (RCTs) showed greater efficacy and less toxicity with ECF [epirubicin, cisplatin, and 5-fluorouracil (5-FU)] [4] or FP4w (5-FU and cisplatin) [5] compared to the traditional FAM (5-FU, adriamycin, and mitomycin) and FAMTX (5-FU, adriamycin, and methotrexate) regimens. ECF became the reference standard in Europe, while FP4w was predominantly adopted in Asia and the United States. Notably, these two reference standards were never directly compared. In 2008, the British REAL-2 non-inferiority RCT demonstrated comparable OS between capecitabine and 5-FU, and between cisplatin and oxaliplatin, when combined with epirubicin [6]. Regarding toxicity, capecitabine and fluorouracil showed similar profiles, while oxaliplatin was linked to lower rates of grade 3–4 neutropenia, alopecia, renal toxicity, and thromboembolism compared to cisplatin, albeit with slightly increased incidences of grade 3–4 diarrhea and neuropathy. This study included patients with locally advanced disease (24.3%), esophageal neoplasia (34.2%), and squamous histology (12.1%), limiting the extrapolation of its data. Conversely, the international non-inferiority RCT ML17032 showed that the combination of 5-FU or capecitabine with cisplatin (FP or CP) were equivalent in activity with different toxicity profile [7]. Vomiting and mucositis were more frequent with FP, whereas hand–foot syndrome and anemia were more common with CP. Various RCTs have indicated that irinotecan and fluoropyrimidine display comparable [8, 9] or superior [10] efficacy to cisplatin and fluoropyrimidine. However, current clinical guidelines place it as an alternative option for patient’s intolerant to platinum [11].

In 2007, the international RCT V325, stood out as the first phase 3 study to show the superior efficacy and quality of life when docetaxel was added to the conventional FP regimen (DCF) [12]. DCF led to a slight increase in response and survival rates, albeit with a significant rise in hematological toxicity. These outcomes were observed in a carefully selected patient group, with 64% having a Karnofsky performance status (PS) of 90–100% and 76% being under 65 years of age. This raised concerns about the broader applicability of DCF beyond the perioperative context [13, 14]. In 2023, data from the French phase 3 PRODIGE 51–GASTFOX RCT revealed that mFLOT/TFOX significantly improved survival compared to FOLFOX (5-FU and oxaliplatin), but with increased incidences of grade 3–4 neuropathy, neutropenia, diarrhea, and fatigue [15]. Subgroup analysis particularly highlighted the benefit of the mFLOT/TFOX triplet in patients with an ECOG–PS of 0 and those with diffuse-type Lauren's cancer. In 2022, the non-inferiority Chinese EXELOX trial established that capecitabine and oxaliplatin (CAPOX) was as effective as the epirubicin, oxaliplatin and capecitabine (EOX) triplet, offered a better safety profile, and enhanced quality of life, thereby challenging the standard practice of adding anthracycline [14, 16]. In a 2017 Cochrane review that included 64 RCTs, the authors conclude that the magnitude of the observed survival benefits with the three-drug regimens is not large enough to be clinically meaningful [3].

Therefore, this background explains why we apparently now have multiple equally efficacious first-line treatment choices for advanced esophagogastric adenocarcinoma, which have not been adequately compared with each other, each characterized by its unique side effect profile. Several systematic reviews have compared alternative regimens to those based on platinum and fluoropyrimidine [14, 17, 18]. However, given the absence of a phase 3 RCT and despite the inherent limitations in such reviews, cisplatin and fluoropyrimidine continues, in the opinion of many, to be the standard treatment and reference regimen in phase 3 trials that include novel molecules, as evidenced by the RCT TOGA with trastuzumab, which precisely used this combination [19]. Contrary to this established paradigm, phase 3 RCTs involving immunotherapy, such as the CHECKMATE-649 [20] combined nivolumab with oxaliplatin and fluoropyrimidine, while the KEYNOTE-859 [21] allowed regimens with either cisplatin or oxaliplatin. Furthermore, the European Medicines Agency (EMA) has approved the use of nivolumab and pembrolizumab for the first-line treatment of advanced HER2-negative esophagogastric adenocarcinoma in combination with a platinum and fluoropyrimidine [20, 22].

Against this backdrop, we have analyzed a national gastric cancer registry to glean insights relevant to this matter. Consequently, our research is focused on assessing and comparing the efficacy and toxicity of four dual-agent chemotherapy regimens, each comprising either cisplatin or oxaliplatin combined with 5-FU or capecitabine. Our aim is to identify the most suitable regimen for broader application in forthcoming RCTs [23].

## Materials and methods

### Study design and population

This study integrated cases from the national, observational, AGAMENON–SEOM registry of the Spanish Society of Medical Oncology, sourced from 40 hospitals. Patients eligible were adults with histologically confirmed advanced, unresectable, or recurrent adenocarcinoma of the distal esophagus, gastroesophageal junction (GEJ), and stomach, without HER2 overexpression who had received first-line chemotherapy doublet with a platinum and a fluoropyrimidine, between 2008 and 2021. Patients who received targeted therapy or immunotherapy in addition to their chemotherapy, or patients with recurrent cancer who had undergone perioperative or adjuvant treatment in the past 6 months were excluded.

This research was conducted in accordance with the Good Clinical Practice guidelines and the Helsinki Declaration and received approval from the Research Ethics Committees of all participating hospitals.

### Therapeutic regimen and variables

The therapeutic regimen, chosen by the medical oncologist, was administered according to the standard clinical practice of the center. Chemotherapy was categorized based on specific platinum and fluoropyrimidine compounds, encompassing combinations such as FOLFOX, FP, CAPOX, and CP. The rationale for selecting each treatment regimen was documented. The Relative Dose Intensity (RDI) was quantified in percentages, defined as the administered dose intensity (drug amount per unit of time, expressed as mg/m^2^ per week) relative to the planned dose for each regimen. In addition, the study collects data on several key parameters: the number of treatment cycles, reasons for discontinuing treatment, and the highest level of toxicity observed, all classified according to the CTCAE v 4.0 (Common Terminology Criteria for Adverse Events).

Baseline variables encompassed patient characteristics (age, sex, ECOG–PS, Charlson comorbidities), tumor characteristics (cancer-related serious complications at diagnosis, location, grade, Lauren classification, presence of signet ring cells, unresectable or metastatic stage, location of metastases and tumor burden), laboratory data and tumor markers [(albumin, bilirubin, alkaline phosphatase, lactate dehydrogenase, hemoglobin, neutrophil-to-lymphocyte ratio (NLR), and carcinoembryonic antigen (CEA)] and whether the primary cancer had been resected.

Treatment effectiveness was assessed using OS and PFS (progression-free survival), defined as the period (months) from the start of the first line until death from any cause (OS), or until progression (PFS), censoring subjects without any event at the last follow-up. The overall response rate (ORR) and disease control rate (DCR), comprising complete response, partial response, and stable disease, were determined based on the RECIST 1.1 criteria through local evaluation.

Data were collected from patients' medical records and recorded via a web tool (http://www.agamenonstudy.com/), which is equipped with filters and enables simultaneous online and telephone-based monitoring.

### Statistical analysis

A basic descriptive analysis was conducted using standard estimators such as means, medians, standard deviations, and percentages. Categorical variables were compared using Chi-square tests. Survival was assessed using the Kaplan–Meier estimator, and survival functions were compared using the log-rank test. Toxicity was evaluated using Amit plots, which include relative risks and their 95% confidence intervals. To analyze prognostic factors, a multivariable Cox proportional hazards (PH) model was utilized. Covariates were selected based on factors previously considered in studies from the AGAMENON–SEOM registry [24, 25]. The PH assumption was verified. Subgroup effects were evaluated using the method proposed by Hahn et al. [26]. According to this author, the estimate in each subgroup is tested against a region of indifference. A fixed sample size approach was employed, conditioned on the number of available patients. This necessitates consideration of the confidence interval magnitudes. A 5% significance level (two-tailed tests) was used for statistical analysis. Statistical analysis was conducted with Stata (version 14.2).

## Results

### Baseline characteristics

At the data cutoff date of December 2021, 4133 patients were registered, 1293 of whom met the inclusion criteria as detailed in Supplementary Fig. 1’s flowchart. Table 1 outlines these patients' baseline characteristics, grouped according to the administered chemotherapy regimen. In this cohort, FOLFOX and CAPOX were each administered to a nearly equal number of patients, together representing 72% of the entire set. Among the cisplatin regimens, the combination with capecitabine was more frequent than with 5-FU, accounting for 20% compared to 8% of the total, respectively.

**Table 1:** Baseline characteristics based on the chemotherapy regimen administered

Baseline characteristics	Total *N* = 1293 (100%)	FOLFOX *N* = 468 (36%)	FP *N* = 107 (8%)	CAPOX *N* = 466 (36%)	CP *N* = 252 (20%)
Age, median (range) < 65 years	66 (20–89)	65 (30–85)	62 (22–82)	69 (31–89)	64 (20–84)
	574 (44.46)	208 (44.64)	62 (57.94)	173 (37.12)	139 (55.16)
Sex, female	429 (33.18)	163 (34.83)	32 (29.91)	160 (34.33)	74 (29.37)
ECOG–PS 0 1 ≥ 2	248 (19.18) 832 (64.35) 213 (16.47)	84 (17.95) 281 (60.04) 103 (22.01)	20 (18.69) 79 (73.83) 8 (7.48)	89 (19.1) 318 (68.24) 59 (12.66)	55 (21.83) 154 (61.11) 43 (17.06)
Charlson comorbidities, > 1	187 (14.46)	72 (15.38)	12 (11.21)	73 (15.67)	30 (11.90)
Cancer-related serious complications at diagnosis^1^	98 (7.58)	44 (9.40)	9 (8.41)	25 (5.36)	20 (7.94)
Primary tumor site Esophagus GEJ Stomach No available	114 (8.82) 148 (11.45) 1014 (78.42) 17 (1.31)	40 (8.55) 62 (13.25) 361 (77.14) 5 (1.07)	16 (14.95) 17 (15.89) 74 (69.16) 0	30 (6.44) 43 (9.23) 385 (82.62) 8 (1.72)	28 (11.11) 26 (10.32) 194 (76.98) 4 (1.59)
Histological grade 1 2 3 No available	129 (9.98) 334 (25.83) 519 (40.14) 311 (24.05)	38 (8.12) 94 (20.09) 228 (48.72) 108 (23.08)	5 (4.67) 34 (31.78) 43 (40.19) 25 (23.36)	45 (9.66) 134 (28.76) 172 (36.91) 115 (24.68)	41 (16.27) 72 (28.57) 76 (30.16) 63 (25)
Lauren type Diffuse and mixed Intestinal No available	535 (41.38) 490 (37.90) 268 (20.73)	228 (48.72) 143 (30.56) 97 (20.73)	44 (41.12) 34 (31.78) 29 (27.1)	176 (37.77) 191 (40.99) 99 (21.24)	87 (34.53) 122 (48.41) 43 (17.06)
Signet ring cells	428 (33.10)	171 (36.54)	38 (35.51)	146 (31.33)	73 (28.97)
Stage, metastasic	1010 (78.11)	361 (77.14)	83 (77.57)	364 (78.11)	202 (80.16)
Metastases sites Peritoneum Liver Liver disease burden > 50%^2^ Ascitis Lung Bone	607 (46.95) 454 (35.11) 95 (20.79) 328 (24.42) 174 (13.46) 136 (10.52)	252 (53.85) 145 (30.98) 22 (14.86) 157 (30.31) 57 (12.18) 72 (15.38)	50 (46.73) 30 (28.04) 8 (26.67) 35 (32.71) 12 (11.21) 8 (7.48)	213 (45.71) 172 (36.91) 40 (23.26) 97 (20.82) 65 (13.95) 40 (8.58)	92 (36.51) 107 (42.46) 25 (23.36) 39 (15.48) 40 (15.87) 16 (6.35)
Number of metastatic sites, > 2	324 (25.06)	104 (22.22)	22 (20.56)	105 (22.53)	93 (36.90)
Primary tumor resected	356 (27.53)	130 (27.78)	31 (28.97)	137 (29.4)	58 (23.02)
Laboratory data Albumin < LLN Bilirubin > ULN ALP > ULN LDH > ULN Hemoglobin < 12 g/dl NLR < 4	314 (24.28) 72 (5.57) 370 (28.62) 292 (22.58) 616 (47.64) 681 (53.54)	141 (30.13) 34 (7.26) 151 (32.26) 111 (23.72) 224 (47.86) 237 (50.97)	31 (28.97) 5 (4.67) 23 (21.50) 17 (15.89) 49 (45.79) 51 (48.57)	93 (19.96) 20 (4.29) 136 (29.18) 100 (21.46) 226 (48.50) 248 (53.91)	49 (19.44) 13 (5.16) 60 (23.81) 64 (25.40) 117 (46.43) 145 (59.92)
Tumor marker, CEA > 10 ng/mL	316 (24.44)	112 (23.93)	21 (19.63)	106 (22.75)	77 (30.56)

*ECOG–PS*, Eastern Cooperative Oncology Group Performance Status;*GEJ*, gastroesophageal junction;*LLL*, low limit normal;*ULN*, upper limit normal; ALP, alkaline phosphatase;*LDH*, lactate dehydrogenase;*NLR*, neutrophil-to-lymphocyte ratio

^1^Cancer-related serious complications at diagnosis include acute liver, respiratory or renal dysfunction, biliary stenosis, intestinal obstruction or pseudo-obstruction, massive ascites, major bleeding, uncontrolled thromboembolic disease, or any other acute medical complication considered to be serious enough by the treating physician.^2^ The liver burden subcategory is based on available data from 476 patients

The median age across all patients was 66 years, with the CAPOX group showing a slightly higher median age of 69, and 63% being 65 years or older. The cohort had twice as many men as women (864 vs. 429). 64% had an ECOG–PS of 1. Among those treated with FOLFOX, 22% (*n* = 114) had an ECOG–PS ≥ 2, compared to only 7.48% (*n* = 8) with FP. Most tumors were in the stomach (78.42%), followed by the GEJ (11.45%) and esophagus (8.82%). 40% (*n* = 519) had a histological grade 3.

Overall, 47% of the patients presented with peritoneal metastases, 35% with hepatic metastases, 24% had ascites, 13% experienced pulmonary metastases, and 10% had bone metastases. In general, patients treated with FOLFOX exhibited a more challenging clinical profile, characterized by a higher prevalence of diffuse and high-grade tumors, poorer functional status, and inferior nutritional parameters.

### Selection and duration of chemotherapy regimen

The most common reason for choosing a chemotherapy regimen was compliance with local protocol (59%) and clinicians' experience (18%). Notably, FOLFOX was the preferred regimen when aiming to maximize the response rate in symptomatic disease (56%, *n* = 24), while CAPOX was predominantly chosen when patient quality of life was a concern (50%, *n* = 48).

The median duration of treatment with cisplatin was 4.14 and 4.17 months for FP and CP regimens, respectively, whereas for oxaliplatin, it was 4.50 and 4.12 months for FOLFOX and CAPOX, respectively. Regarding capecitabine, it was administered for 4.62 months in CP and 4.60 months in CAPOX; conversely, 5-FU was given for 4.27 months in FP and 5.40 months in FOLFOX.

64% of patients (*n* = 827) received at least 80% of the RDI of fluoropyrimidine, 55% and 72% for capecitabine regimens (CP and CAPOX, respectively), and 60% and 73% for 5-FU regimens (FOLFOX and FP, respectively). Regarding platinum-based therapies, 60% (*n* = 780) reached 80% of RDI, with 48% and 66% for cisplatin (CP and FP, respectively) and 56% and 71% for oxaliplatin (FOLFOX and CAPOX, respectively).

Only 20% of the patients (*n* = 253) underwent platinum treatment for more than 180 days, with the distribution being 5% for cisplatin (FP), 5% for cisplatin (CP), 9% for oxaliplatin (CAPOX) and 26% for oxaliplatin (FOLFOX). Conversely, 35% (*n* = 451) exceeded 180 days of fluoropyrimidine treatment, 16% and 41% with 5FU (FP and FOLFOX, respectively), and 31% and 35% with capecitabine (CP and CAPOX, respectively).

The primary reason for discontinuing platinum was cancer progression, accounting for 43% and 49% for cisplatin (FP and CP, respectively) and 42% and 43% for oxaliplatin (CAPOX and FOLFOX, respectively). Completion of the treatment plan was the next most common cause for discontinuing cisplatin (28% for CP and 39% for FP), while for oxaliplatin, it was toxicity (27% for CAPOX and 29% for FOLFOX). Regarding fluoropyrimidines, cancer progression was the main reason for discontinuation both for capecitabine (66% and 71%, CAPOX and CP, respectively) and for 5-FU (56% and 60%, FP and FOLFOX, respectively). These data are detailed for each drug according to the regimen used in Supplementary Table 1.

### Efficacy

**Fig. 1:**
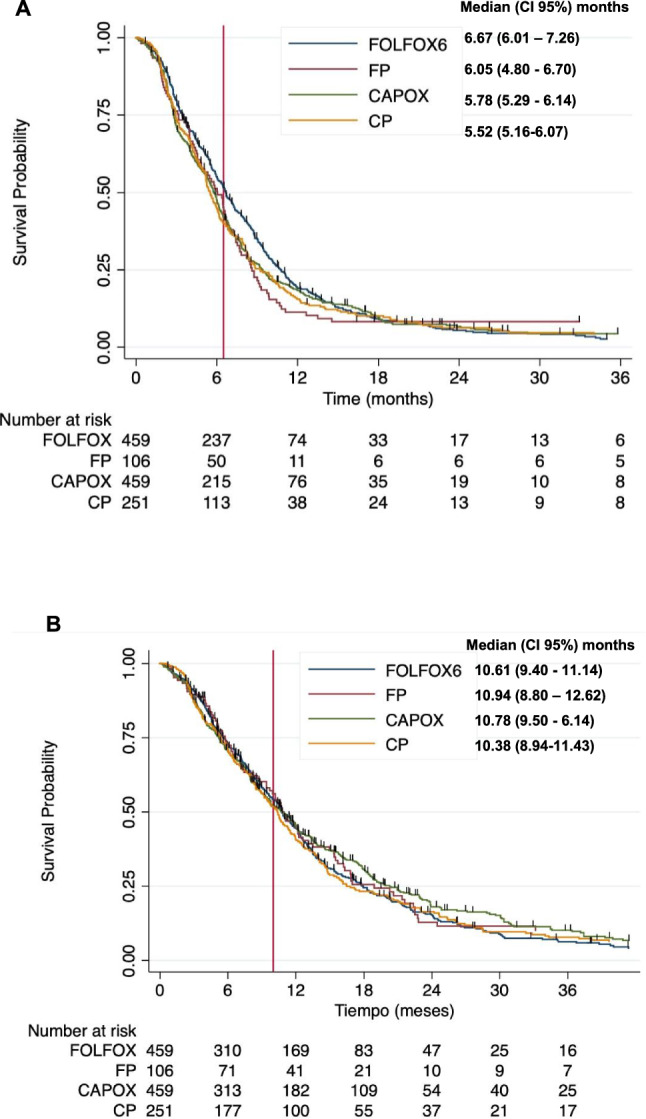
Survival curves according to chemotherapy regimen: PFS (**A**) and OS (**B**)

**Fig. 2:**
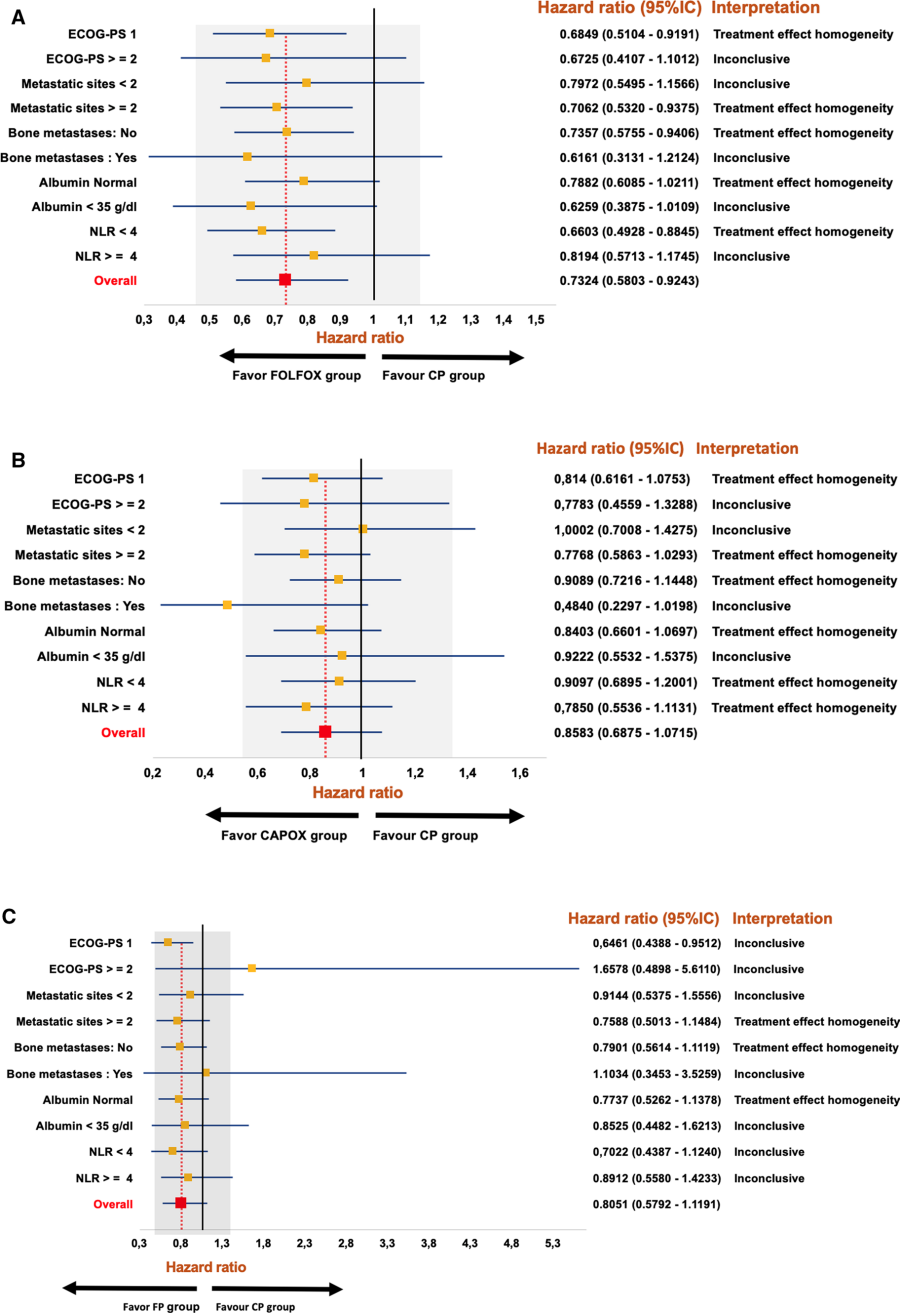
Forest plot of hazard ratio (HR) estimates for PFS in subgroup interactions by scheme FOLFOX (**A**), CAPOX (**B**) and FP (**C**). The red dotted vertical line highlights the overall treatment effect point (or main effect) for every scheme compared to CP (reference). The area shaded in gray represents the ‘indifference zone’ for the overall treatment effect, assuming that treatment effects between 80 and 125% of the 95% CI for the main effect do not represent clinically meaningful differences between each subgroup and the main effect. All subgroups with 95% CI that are only compatible with values within the indifference zone show treatment effect homogeneity. Subgroups with 95% CI that do not overlap with the red dotted vertical line (main effect) show evidence of treatment effect heterogeneity. All other subgroups are inconclusive. The 95% CI for the overall treatment effect HR is 0.5803–0.9243 corresponding to an indifference zone (shaded in grey) of 0.4642–1.1554. Abbreviations: ECOG–PS, Eastern Cooperative Oncology Group Performance Status; NLR, neutrophil-to-lymphocyte ratio; CI confidence interval. The 95% CI for the overall treatment effect HR is 0.6875–1.0715 corresponding to an indifference zone (shaded in grey) of 0.55–1.3394. Abbreviations: ECOG–PS, Eastern Cooperative Oncology Group Performance Status; NLR, neutrophil-to-lymphocyte ratio; CI confidence interval. The 95% CI for the overall treatment effect HR is 0.5792–1.1191 corresponding to an indifference zone (shaded in grey) of 0.4634–1.3989. Abbreviations: ECOG–PS, Eastern Cooperative Oncology Group Performance Status; NLR, neutrophil-to-lymphocyte ratio; CI confidence interval

At the time of this analysis, there were 1,167 recorded progression events, representing 90% of cases, with a median PFS of 6.01 months (95% CI 5.72–6.30). In addition, there were 1,075 death events, accounting for 83% of cases, with a median OS of 10.67 months (95% CI 10.18–11.27). Kaplan–Meier curves, categorized by treatment regimen, are depicted in Fig. 1. The log-rank test, when applied to these stratified data, failed to reject the null hypothesis of no significant differences between regimens (log-rank test, *p* value = 0.23 for PFS, and 0.51 for OS, respectively). To clarify comparative therapeutic efficacy, we fitted Cox proportional hazards (PH) regression models for PFS and OS, considering the treatment regimens. The model for PFS identified several factors associated with a poorer prognosis, including worse ECOG–PS, presence of more than two metastatic sites, bone metastases, hypoalbuminemia, and increased NLR (Table 2). Notably, the use of FOLFOX was associated with improved PFS (HR 0.73; 95% CI 0.58–0.92, p = 0.009). However, its impact on OS was more limited, and the estimate was noisy, not allowing for the rejection of the null hypothesis of no effect (HR 0.90; 95% CI 0.71–1.15, p = 0.402, as shown in Supplementary Table 2) compared to CP. Similarly, in a sensitivity analysis focusing on regimens containing oxaliplatin (namely CAPOX or FOLFOX), the null hypothesis (that oxaliplatin regimens are equivalent to cisplatin regimens in terms of OS or PFS) also could not be rejected, possibly due to the relatively modest effect of CAPOX treatments (HR 0.85, 95% CI 0.71–1.01, *p* value = 0.077, as shown in Supplementary Table 3). Subgroup analysis revealed no statistical evidence of heterogeneous effects, indicating a consistent benefit with FOLFOX across all analyzed strata (Fig. 2). In addition, no interactions were observed when grouping by chemotherapy regimen, nor among platinum-based regimens (Supplementary Table 4 and 5). Regarding cisplatin regimens, the data do not allow for the rejection of the null hypothesis that CP and FP are similar in terms of PFS. Although there were indications that FP might be superior to CP in patients with an ECOG–PS of 0, the evidence was inconclusive. The interaction between the therapeutic effect and time was tested, finding no evidence that the year of initiation of first-line therapy altered the comparative effect of FOLFOX.

**Table 2:** Cox proportional hazards regression for PFS

Covariates	HR	95% CI HR	*p* value
Age	0.9986	0.9912–1.0061	0.713
Sex Male Female	Ref 1.0187	Ref 0.8550–1,2137	– 0.836
ECOG–PS 0 1 ≥ 2	Ref 1.2664 1.8492	Ref 1.0285–1.5594 1.4012–2.4404	– **0.026** **0.000**
Primary tumor site Stomach Esophagus GEJ	Ref 0.9395 1.2751	Ref 0.6832–1.2920 0.9897–1.6430	– 0.701 0.060
Lauren Intestinal Diffuse Mixed	Ref 1.0780 0.9521	Ref 0.8782–1.3232 0.6530–1.3882	– 0.473 0.799
Histological grade 1 2 3	Ref 1.2330 1.2770	Ref 0.9519–1.5971 0.9683–1.6841	– 0.113 0.083
Metastatic sites < 2 ≥2	Ref 1.2292	Ref 1.0410–1.4514	– **0.015**
Ascitis No Yes	Ref 1.1797	Ref 0.9580–1.4528	– 0.120
Bone metastases No Yes	Ref 1.4600	Ref 1.1285–1.8887	– **0.004**
*Albumin* Normal < 35 g/dL	Ref 1.2811	Ref 1.0554–1.5549	– **0.012**
NLR	1.0234	1.0051–1.0420	**0.012**
Chronic cardiopathy No Yes	Ref 1.0277	Ref 0.7123–1.4827	– 0.884
Charlson comorbidities < 2 ≥ 2	Ref 0.9227	Ref 0.6594–1.2910	– 0.639
Chemotherapy regimen CP FOLFOX CAPOX FP	Ref 0.7324 0.8583 0.8051	Ref 0.5803–0.9243 0.6875–1.0715 0.5792–1.1191	Ref **0.009** 0.177 0.197

The bold *p*-values indicate statistical significance (*p* < 0.05)

*ECOG–PS*, Eastern Cooperative Oncology Group Performance Status;*GEJ*, gastroesophageal junction;* LLL*, low limit normal;*NLR*, neutrophil-to-lymphocyte ratio;*HR*, hazard ratio;*CI* confidence interval

The ORR across the entire sample was 31.56% and the DCR was 79.82%. These rates were consistent across the various chemotherapy regimens (*p* = 0.181 for ORR and *p* = 0.127 for DCR, respectively, as detailed in Table 3).

**Table 3:** Tumor response by RECIST 1.1 criteria through local evaluation

	Total *N* = 1293 (100%)	FOLFOX6 *N* = 468 (36%)	FP *N* = 107 (8%)	CAPOX *N* = 466 (36%)	CP *N* = 252 (19%)	*P* (X^2^ test)
ORR 95% CI	31.56 (28.85–34.36)	31.19 (26.70–35.96)	37.11 (27.52–47.52)	29.15 (24.73–33.88)	34.07 (27.92–40.65)	0.181
DCR, 95% CI	79.82 (77.36–82.13)	83.17 (79.16–86.69)	83.51 (74.60–90.27)	77.14 (72.69–81.17)	76.99 (70.95–82.31)	0.127

*ORR*, Overall Response Rate;* DCR*, Disease Control Rate

### Toxicity

Common adverse events observed across all grades in all regimens included fatigue, emesis, anemia, and neutropenia, with neuropathy specifically noted in oxaliplatin-based regimens (Table 4). In terms of grade 3–4 toxicity, neutropenia was most common: FP (28.04%), FOLFOX (27.11%), CP (16.27%), and CAPOX (7.39%). Higher instances of grade 3–4 renal toxicity were observed in cisplatin regimens, with FP at 2.80% and CP at 1.59%, compared to CAPOX (0.43%) and FOLFOX (0.00%). Conversely, grade 3–4 neuropathy was lower in CP (0.00%) and FP (0.93%) than in CAPOX (4.13%) and FOLFOX (7.16%). The incidence of hand–foot syndrome was more prevalent in regimens containing capecitabine, with CP at 45.63% and CAPOX at 32.26%, vs. 5-FU in FOLFOX (11.28%) and FP (7.48%), although the occurrence of grade 3–4 hand–foot syndrome was less than 2% in all regimens. FP showed a higher rate of grade 3–4 mucositis (4.76%), and CP had a higher incidence of grade 3–4 thrombosis (5.95%) compared to other regimens. The rates of hospitalization due to toxicity were as follows: FP (24.04%), FOLFOX (20.14%), CAPOX (19.81%), and CP (17.48%).

**Table 4:** Treatment-related toxicity by chemotherapy regimen

Toxicity (*N* = 1280)	FOLFOX (*n* = 461)		FP (*n* = 107)		CAPOX (*n* = 460)		CP (*n* = 252)	
	Total (%)	Grade 3–4 (%)	Total (%)	Grade 3–4 (%)	Total (%)	Grade 3–4 (%)	Total (%)	Grade 3–4 (%)
Anemia	65.73	5.64	57.94	4.67	59.78	4.57	60.71	5.56
Neutropenia	52.06	27.11	55.14	28.04	30.65	7.39	45.63	16.27
Febrile neutropenia	5.21		3.74		2.17		5.16	
Thrombocytopenia	31.45	1.30	22.43	0.93	23.70	3.48	13.89	1.98
Emesis	60.52	2.60	57.01	0.93	55.43	3.91	59.13	6.35
Diarrhea	39.48	3.90	38.32	2.80	40.00	5.65	28.57	4.37
Stomatitis	33.41	1.52	42.99	4.67	27.17	1.52	22.22	0.79
Fatigue	76.57	8.03	71.96	4.67	67.83	8.70	59.52	6.75
Hand–foot syndrome	11.28	0.65	7.48	0.93	32.26	1.96	45.63	1.59
Neuropathy	68.76	7.16	16.82	0.93	62.61	4.13	28.17	0.00
Alopecia	5.42	1.74^1^	23.36	2.80^1^	3.91	1.30^1^	7.54	0.79^1^
Hyperbilirubinemia	7.38	1.74	2.80	0.00	6.74	1.09	3.97	1.19
Increased aspartate aminotransferase	17.14	1.74	11.21	0.93	13.07	0.22	6.75	0.79
Renal toxicity	4.99	0.00	9.35	2.80	6.52	0.43	9.52	1.59
Venous thromboembolic disease	7.59	3.47	12.15	3.74	7.17	3.70	11.11	5.95
Heart failure	1.52	0.65	0.93	0.00	0.65	0.43	1.19	1.19
Toxicity-related hospital admission	20.14		24.04		19.81		17.48	

^1^ Grade 3 (Grade 4 not applicable for alopecia according to CTCAE v 4.0)

Considering the nuanced differences in toxicity profiles across the various drug regimens, a direct comparison was undertaken (refer to Amit plot in Supplementary Fig. 2). Patients receiving oxaliplatin were found to have a lower risk of thrombotic events (RR = 0.64, 95% CI 0.44–0.95, *p* = 0.026), renal toxicity (RR = 0.39, 95% CI 0.17–0.89, *p* = 0.021), and neutropenia (RR = 0.78, 95% CI 0.66–0.92, *p* = 0.004) compared to those treated with cisplatin. On the other hand, oxaliplatin was associated with a higher risk of peripheral neuropathy (RR = 6.50; 95% CI 3.91–10.78; *p* < 0.001), thrombocytopenia (RR = 1.75; 95% CI 1.10–2.80; *p* = 0.016), and diarrhea (RR = 1.40; 95% CI 1.02–1.91; *p* = 0.035). Anemia (RR = 0.76; 95% CI 0.63–0.91; *p* = 0.003), stomatitis (RR = 0.60, 95% CI 0.42–0.87, *p* = 0.016), and neutropenia (RR = 0.57; 95% CI 0.48–0.67; *p* < 0.001) were less prevalent in patients treated with capecitabine compared with 5-FU, while hand–foot syndrome (HFS) was more common in those receiving capecitabine (RR = 5.13; 95% CI 2.75–9.62; *p* < 0.001).

## Discussion

The cisplatin–5FU regimen, initially established in the 1980s [27], and subsequently refined over time, has emerged as the benchmark for treating advanced esophagogastric adenocarcinoma, a status affirmed by numerous randomized controlled trials (RCTs) [20, 22, 28–33]. The integration of novel agents into this classical regimen is an active area of research, emphasizing the importance of investigating combined efficacy, synergies, and additive toxicities. This knowledge is crucial for optimizing treatment protocols and enhancing clinical outcomes.

In our research, we assessed the efficacy and toxicity profiles of four platinum and fluoropyrimidine-based doublet chemotherapy regimens in first-line treatment for advanced HER2-negative esophagogastric adenocarcinoma, drawing upon data from the AGAMENON–SEOM national esophagogastric cancer registry. The results demonstrate comparable activity in terms of ORR across all chemotherapy schemes. FOLFOX was associated with improved PFS of 6.67 months (HR 0.73; 95% CI 0.58–0.92, *p* = 0.009) but not OS of 10.61 months (HR 0.90; 95% CI 0.71–1.15, *p* = 0.045) compared to CP, with PFS of 5.52 months and OS of 10.38 months, showing a consistent effect across all subgroups. This difference was not found between the two oxaliplatin-based regimens, FOLFOX and CAPOX, nor between the two cisplatin-based regimens, FP and CP. Each regimen was associated with specific toxicity profiles: FOLFOX had higher rates of asthenia (77%) and neuropathy (69%); neutropenia was significant in FOLFOX and FP (52% and 55%, respectively); hand–foot syndrome was prominent in CP (46%); and more thromboembolic events were noted in cisplatin-based schemes, FP and CP (12% and 11%, respectively). There were no differences in emesis, and diarrhea was slightly lower with CP. These findings suggest that selecting a chemotherapy regimen should consider its toxicity profile to improve treatment tolerance, continuity, and patient quality of life.

Our data align with previous literature in indicating that regimens containing oxaliplatin, particularly FOLFOX, outperform those with cisplatin, and contribute to reinforcing this evolving hypothesis [34]. In a study by the German AIO group, FOLFOX was found to have lower toxicity compared to FP, suggesting enhanced efficacy in older adults [35]. Further supporting this, the REAL2 RCT's secondary analysis showed EOX outperforming ECF in OS, 11.2 vs. 9.9 months (HR 0.80, 95% CI 0.66–0.97; *p* = 0.02), without significant differences in PFS and ORR among the regimens [6]. Notably, compared to cisplatin, oxaliplatin was associated with less frequent occurrences of severe neutropenia, alopecia, renal toxicity, and thromboembolism, though it showed a modest increase in severe diarrhea and neuropathy. Consistently, the Serbian Oncology and Radiology Institute's RCT comparing FOLFOX with FP favored the oxaliplatin regimen in terms ORR and OS, with a longer time to progression. However, the result was borderline significant (*p* = 0.073) [36]. This RCT also reported higher rates of severe hematologic and gastrointestinal toxicities with FP. Taken together, the data point to the superiority of oxaliplatin, with a meta-analysis including these three studies, all from 2008, highlighting a modest survival advantage over cisplatin (HR for death 0.88; 95% CI 0.78–0.99).

[18]. An earlier analysis of the AGAMENON–SEOM registry, focusing on HER2-positive tumors, found comparable results with ToGA and CAPOX–trastuzumab regimens, while suggesting a potential advantage of FOLFOX–trastuzumab, particularly for those subtypes that typically exhibit less sensitivity to trastuzumab. However, this potential benefit requires further validation through RCTs [37].

From 2010 to 2019, several RCTs attempted to improve the effectiveness of platinum–fluoropyrimidine doublets by incorporating targeted drugs, but these efforts did not yield favorable results [28–31]. In contemporary trials, there's a notable trend of equating all platinum and fluoropyrimidine doublets under the assumption that any differences between existing options are thought to be minimal. As a result, the selection of a chemotherapy regimen has commonly been based on toxicity, considering the profile of the experimental drug [31, 38] the potential interaction with the immune system, or dosing schedules (bi-weekly or tri-weekly) to enhance efficacy and tolerance when combined with new drugs.

In the phase 3 RCT CHECKMATE-649, oxaliplatin-based regimens, FOLFOX and CAPOX, were tested alongside nivolumab administered either bi-weekly or tri-weekly [20]. Oxaliplatin was preferred over cisplatin due to its potentially better toxicity profile. Among patients with PD-L1 ≥ 5, there was no significant difference in OS when comparing different chemotherapy combinations. Specifically, OS was 14.3 months for FOLFOX with nivolumab versus 11.3 months for FOLFOX alone (HR 0.71, 0.57–0.88), and 15 months for CAPOX with nivolumab versus 11 months for CAPOX alone (HR 0.69, 0.55–0.85), *p* = 0.9. The phase 3 RCT KEYNOTE-859 compared tri-weekly chemotherapy regimens, CAPOX (86% of the sample) or FP, each combined with pembrolizumab, finding no differences in activity between both in subgroup analysis [21]. Finally, a network meta-analysis involving eight phase 3 RCTs examined the efficacy and safety of PD-1 inhibitors combined with either oxaliplatin- or cisplatin-based chemotherapy as first-line treatment for advanced gastric cancer [39]. In tumors with a CPS ≥ 1, oxaliplatin combinations showed enhanced efficacy (HR 0.75, 0.57–0.99). PFS was more prolonged with oxaliplatin compared to cisplatin (HR 0.72, 0.53–0.99), with no significant difference in ORR (RR 1.09, 0.74–1.61). This clinical observation was suggested to potentially stem from the stronger immunogenic cell death effect of oxaliplatin. The rate of severe side effects was comparable between both regimens (RR 0.86, 0.66–1.12). However, these results are preliminary and primarily hypothesis-generating, as only two of the RCTs in the meta-analysis were international, and out of 5723 patients studied, only 250 were treated with cisplatin in these trials.

The phase 3 SPOTLIGHT and GLOW RCTs combined oxaliplatin-based regimens, FOLFOX and CAPOX, with zolbetuximab (anti-CLDN18.2 +) [33]. This choice could be due to zolbetuximab's high emetogenic profile, making its combination with cisplatin less advisable. Our analysis validates this approach, revealing a higher incidence of emesis with cisplatin-based regimens compared to those with oxaliplatin. The SPOTLIGHT RCT showed an OS of 18.23 months with the addition of zolbetuximab versus 15.54 months with FOLFOX (HR 0.75, *p* = 0.0053). The GLOW RCT reported an OS of 14.39 months in the zolbetuximab plus CAPOX group, compared to 12.16 months with CAPOX alone (HR 0.77, *p* = 0.0118). While not directly comparable studies, FOLFOX achieved better results both in monotherapy and combined with zolbetuximab.

The trends shown here occur while controlling for multiple covariates with already elucidated prognostic effects. An ECOG–PS > 1, more than two metastatic locations, the presence of ascites, bone metastases, hypoalbuminemia, and a raised NLR were identified as poor prognostic factors without distinct subgroup effects linked to any covariate among the studied chemotherapy regimens. These factors have been previously described in works from this registry [24], with a particular emphasis on their significance in HER2-positive tumors [37], a conclusion that is supported by prior meta-analyses in this field [40].

Other relevant factors for future research consideration are infusion times and the direct and indirect costs of different regimens. Protocols that include capecitabine do not require hospital day time due to oral administration, and oxaliplatin has shorter administration time than cisplatin, avoiding the pre- and post-hydration needed to prevent tubulopathy. Furthermore, the economic impact of toxicity, such as costs from hospitalizations or emergency visits due to adverse events, can influence the overall cost of each regimen. In our analysis, around 20% of patients in each group were hospitalized due to toxicity, with the FP regimen showing the highest rate (24%) and CP the lowest (17%).

The most notable limitation of this study is its retrospective nature. Progression and mortality are endpoints often reliably recorded in medical records, whereas toxicity is a more nuanced endpoint. It is subject to greater uncertainty in retrospective studies and may be influenced by variability in data collection among different investigators and centers. Second, the choice of chemotherapy treatment and the timing of computed tomography scans were based on individual center criteria. Finally, it is crucial to note that our series does not include HER2-positive cases or chemotherapy combinations with other agents. Therefore, we cannot assess how our findings might compare with regimens combined with trastuzumab, immunotherapy, or a biological agent.

In conclusion, the AGAMENON–SEOM series data, encompassing 1293 patients with advanced HER2-negative esophagogastric adenocarcinoma, reveals that FOLFOX is superior in PFS compared to CP. The adverse effect profiles of the platinum-based regimens differ, with neuropathy more prevalent in oxaliplatin and thromboembolic events more common in cisplatin.

### Supplementary Information

Below is the link to the electronic supplementary material.

Supplementary file1 (DOCX 35 KB)

Supplementary file2 (DOCX 81 KB)

Supplementary file3 (DOCX 18 KB)

Supplementary file4 (DOCX 17 KB)

Supplementary file5 (DOCX 16 KB)

Supplementary file6 (DOCX 16 KB)

Supplementary file7 (DOCX 15 KB)

## Data Availability

Available upon request to the authors.
