# Supplementary material for: Is there a preferred platinum and fluoropyrimidine regimen for advanced HER2-negative esophagogastric adenocarcinoma? Insights from 1293 patients in AGAMENON–SEOM registry
Source: Clin Transl Oncol. 2024 Feb 15;26(7):1674–86. doi: 10.1007/s12094-024-03388-6 (PMC11178610; doi:10.1007/s12094-024-03388-6)
Supplement: Supplementary file 1 — Supplementary file1 (DOCX 35 KB) [file 12094_2024_3388_MOESM1_ESM.docx]

**Annex Figure 1.** Flowchart.

- Untreated or monochemotherapy (n=**811**)
- HER2+ (**n=743**) or unknown (**n=91**)

Period: January 1, 2008 to December 14, 2021

Total (n=**4133**)

Tiem

for eligibility (n=2169)

Polichemotherapy (n=**2488**)

.

- Regimen based on 3 cytotoxic agents (n=**802**)

Regimen based on 2 cytotoxic agents (n=**1686**)

- Regimen based on docetaxel or irinotecan (n=**134**)
- Other regimens (n=**126**)

Regimen based on platinum and fluoropyrimidine (n=**1426**)

- Non-standard or modified regimens (n=**133**)

Met eligibility criteria for this analysis (n=**1293**)
