# Supplementary figures and images for: Is there a preferred platinum and fluoropyrimidine regimen for advanced HER2-negative esophagogastric adenocarcinoma? Insights from 1293 patients in AGAMENON–SEOM registry
Source: Clin Transl Oncol. 2024 Feb 15;26(7):1674–86. doi: 10.1007/s12094-024-03388-6 (PMC11178610; doi:10.1007/s12094-024-03388-6)

**Annex Figure 2.** Amit plot of grade 2-4 toxicity by platinum (A) and fluoropirimidine (B).

**
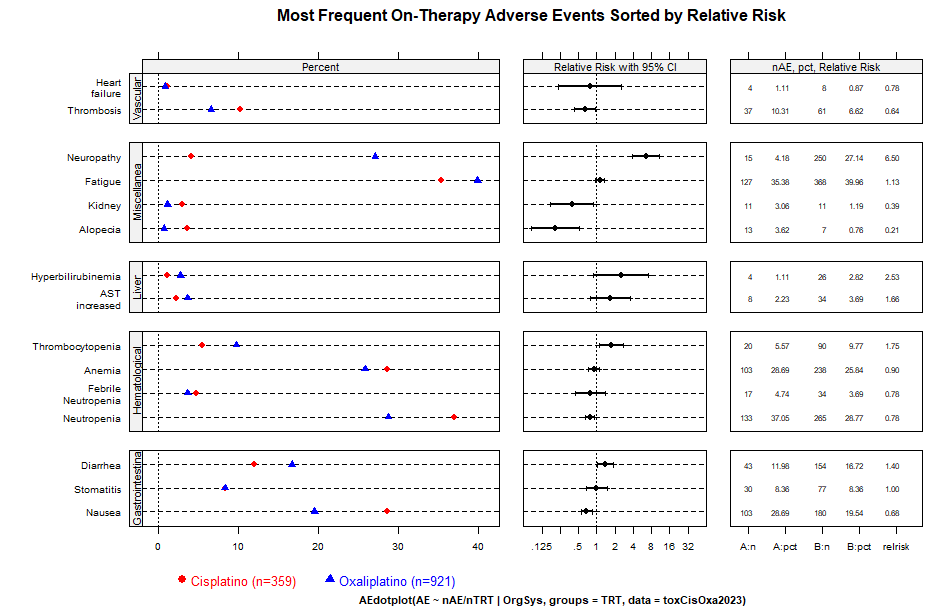
**

**A**


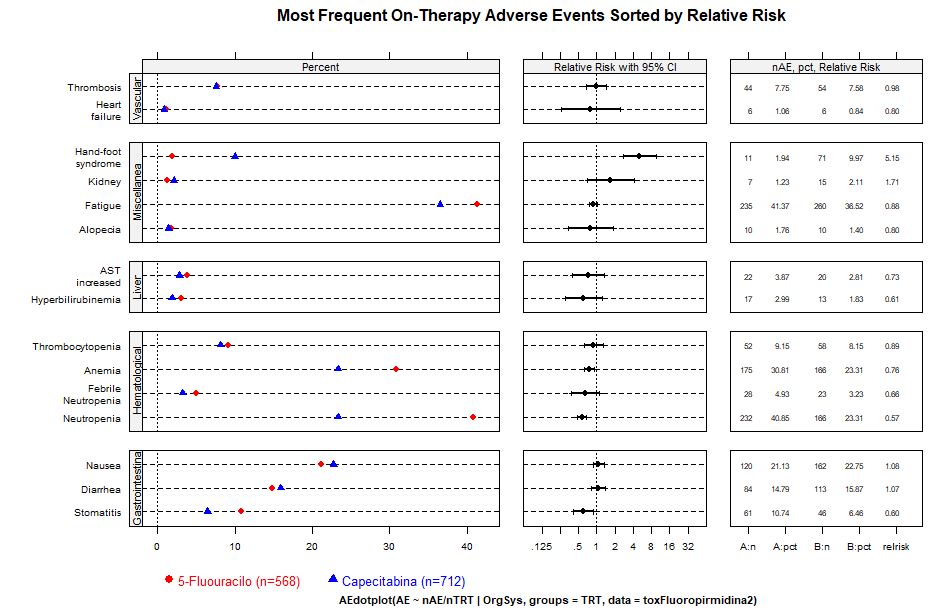


**B**

.

Supplement: Supplementary file 2 — Supplementary file2 (DOCX 81 KB) [file 12094_2024_3388_MOESM2_ESM.docx]
