# Supplementary material for: Is there a preferred platinum and fluoropyrimidine regimen for advanced HER2-negative esophagogastric adenocarcinoma? Insights from 1293 patients in AGAMENON–SEOM registry
Source: Clin Transl Oncol. 2024 Feb 15;26(7):1674–86. doi: 10.1007/s12094-024-03388-6 (PMC11178610; doi:10.1007/s12094-024-03388-6)
Supplement: Supplementary file 3 — Supplementary file3 (DOCX 18 KB) [file 12094_2024_3388_MOESM3_ESM.docx]

**Annex Table 1.** Treatment duration RDI, and reason for choosing or discontinuing regimens or drugs.

|  | **FOLFOX** | | **FP** | | **CAPOX** | | **CP** | |
| --- | --- | --- | --- | --- | --- | --- | --- | --- |
|  | **Oxaliplatin** | **5-FU** | **Cisplatin** | **5-FU** | **Oxaliplatin** | **Capecitabine** | **Cisplatin** | **Capecitabine** |
| **Treatment duration**  **(median, months)** | 4.50 | 5.40 | 4.14 | 4.27 | 4.12 | 4.60 | 4.17 | 4.62 |
| **Reason for choosing the regimen**  *Compliance with local protocol (n= 760)*  *Clinicians' experience* (n= 229)  *Patient comorbidities (n= 55)*  *Quality of life (n=96)*  *Response maximization in symptomatic (n=43)*  *Inability of the patient to ingest (n=12)*  *Other (n= 98)* | 247 (32.50%)  86 (37.55%)  25 (45.45%)  38 (39.58%)  24 (55.81%)  10 (83.33%)  38 (38.76%) | | 54 (7.11%)  24 (10.48%)  4 (7.27%)  3 (3.12%)  4 (9.30%)  2 (16.67%)  16 (16.34%) | | 268 (35.26%)  90 (39.30%)  23 (41.82%)  48 (50%)  14 (32.56%)  0 (0.00%)  23 (23.47%) | | 191 (25.13%)  29 (12.66%)  3 (5.45%)  7 (7.29%)  1 (2.33%)  0 (0.00%)  21 (21.43%) | |
| **Treatment time**  **> 180 days (%)** | 25.95 | 40.77 | 4.67 | 16.82 | 19.40 | 35.34 | 14.68 | 31.35 |
| **RDI > 80% (%)** | 56.22 | 59.66 | 66.36 | 72.90 | 70.47 | 71.55 | 47.62 | 55.16 |
| **Reason for**  **discontinuation (%)**  *Toxicity*  *Progression*  *CPT* | 29.00  42.69  15.98 | 9.22  60.05  14.66 | 9.52  43.81  39.05 | 6.93  56.44  28.71 | 26.97  42.32  21.05 | 12.64  65.75  10.57 | 16.33  49.40  28.29 | 10.48  71.37  10.89 |

Abbreviations: RDI; Relative Dose Intensity; CPT; Completed planned treatment
