# Supplementary material for: Is there a preferred platinum and fluoropyrimidine regimen for advanced HER2-negative esophagogastric adenocarcinoma? Insights from 1293 patients in AGAMENON–SEOM registry
Source: Clin Transl Oncol. 2024 Feb 15;26(7):1674–86. doi: 10.1007/s12094-024-03388-6 (PMC11178610; doi:10.1007/s12094-024-03388-6)
Supplement: Supplementary file 4 — Supplementary file4 (DOCX 17 KB) [file 12094_2024_3388_MOESM4_ESM.docx]

**Annex Table 2.** Cox proportional hazards regression for OS.

| **Covariates** | **HR** | **95% CI HR** | **p-value** |
| --- | --- | --- | --- |
| **Age** | 1.0016 | 0.9937 - 1.0096 | 0.691 |
| **Sex,** male  Female | Ref.  0.9588 | Ref.  0.7970 - 1.1534 | -  0.655 |
| **ECOG-PS,** 0  1  ≥2 | Ref.  1.2674  2.2119 | Ref.  1.0168 - 1.5796  1.6492 - 2.9668 | -  **0.035**  **0.000** |
| **Primary tumor site,** stomach  Esophagus  GEJ | Ref.  1.152904  1.1733 | Ref.  0.8307 - 1.6000  0.8929 - 1.5417 | -  0.395  0.251 |
| **Lauren,** intestinal  Diffuse  Mixed | Ref.  1.2596  0.9593 | Ref.  1.0113 - 1.5689  0.6458 - 1.4250 | -  **0.039**  0.837 |
| **Histological grade,** 1  2  3 | Ref.  1.2207  1.1618 | Ref.  0.9305 - 1.6014  0.8665 - 1.5577 | -  0.150  0.316 |
| **Metastatic sites,** <2  > 2 | Ref.  1.3378 | Ref.  1.1227 - 1.5942 | -  **0.001** |
| **Ascitis,** no  Yes | Ref.  1.2502 | Ref.  1.0048 - 1.5556 | -  **0.045** |
| **Bone metastases,** no  Yes | Ref.  1.3062 | Ref.  0.9929 - 1.7185 | -  0.056 |
| **Albumin,** normal  < 35 g/dL | Ref.  1.4193 | Ref.  1.1573 - 1.7407 | -  **0.001** |
| **NLR** | 1.0225 | 1.0042 - 1.0411 | **0.016** |
| **Chronic cardiopathy,** no  Yes | Ref.  0.9642 | Ref.  0.6712 - 1.3849 | -  0.843 |
| **Charlson comorbidities,** <2  > 2 | Ref.  0.9476 | Ref.  0.6778 - 1.3249 | -  0.753 |
| **Chemotherapy regimen,** CP  FOLFOX  CAPOX  FP | Ref.  0.9031  0.8453  0.7861 | Ref.  0.7114 - 1.1464  0.6715 - 1.0640  0.5581 - 1.1071 | -  0.402  0.152  0.168 |

Abbreviations: ECOG-PS, Eastern Cooperative Oncology Group Performance Status; NLR, neutrophil-to-lymphocyte ratio; HR, hazard ratio; CI, confidence interval.
