# Supplementary material for: Is there a preferred platinum and fluoropyrimidine regimen for advanced HER2-negative esophagogastric adenocarcinoma? Insights from 1293 patients in AGAMENON–SEOM registry
Source: Clin Transl Oncol. 2024 Feb 15;26(7):1674–86. doi: 10.1007/s12094-024-03388-6 (PMC11178610; doi:10.1007/s12094-024-03388-6)
Supplement: Supplementary file 5 — Supplementary file5 (DOCX 16 KB) [file 12094_2024_3388_MOESM5_ESM.docx]

**Annex Table 3**. Cox proportional hazards regression analysis for PFS by platin-based regimen.

| **Covariates** | **HR** | **95% CI, HR** | **p-value** |
| --- | --- | --- | --- |
| **Age** | 0.9990 | 0.9917 – 1.0064 | 0.799 |
| **Sex,** male  Female | Ref  1.0365 | Ref  0.8710 – 1.2335 | -  0.686 |
| **ECOG-PS,** 0  1  ≥2 | Ref  1.2499  1.8465 | Ref  1.0154- 1.5386  1.4004 – 2.4347 | -  **0.035**  **0.000** |
| **Primary tumor site,** stomach  Esophagus  GEJ | Ref  0.9115  1.2351 | Ref  0.6638 - 1.2516  0.9600 - 1.5891 | -  0.567  0.100 |
| **Lauren,** intestinal  Diffuse  Mix | Ref  1.0703  0.9219 | Ref  0.8712 – 1.3149  0.6335 – 1.3415 | -  0.518  0.671 |
| **Histological grade,** 1  2  3 | Ref  1.2180  1.2293 | Ref  0.9446 – 1.5704  0.9347 - 1.6167 | -  0.128  0.140 |
| **Metastatic sites,** < 2  >2 | Ref.  1.2396 | Ref.  1.0504 - 1.4629 | -  **0.011** |
| **Ascitis,** no  Yes | Ref.  1.1382 | Ref.  0.9275 - 1.3968 | -  0.215 |
| **Bone metastases,** no  Yes | Ref.  1.4383 | Ref.  1.1122 – 1.8599 | -  **0.006** |
| **Albumin,** normal  < 35g/dL | Ref.  1.2341 | Ref.  1.0207 - 1.4921 | **-**  **0.030** |
| **NLR** | 1.0227 | 1.0046 – 1.0412 | **0.014** |
| **Chronic cardiopathy,** no  Yes | Ref.  1.0781 | Ref.  0.7491 - 1.5517 | -  0.685 |
| **Charlson comorbidities,** <2  >2 | Ref.  0.9016 | Ref.  0.6446 - 1.2611 | -  0.545 |
| **Platinum-based regimen,** Cisplatin - based  Oxaliplatin-based | Ref.  0.8508 | Ref.  0.7113 – 1.018 | -  0.077 |

Abbreviations: ECOG-PS, Eastern Cooperative Oncology Group Performance Status; GEJ, gastroesophageal junction; LLL, low limit normal; NLR, neutrophil-to-lymphocyte ratio; HR, hazard ratio; CI, confidence interval.
