# Supplementary material for: Is there a preferred platinum and fluoropyrimidine regimen for advanced HER2-negative esophagogastric adenocarcinoma? Insights from 1293 patients in AGAMENON–SEOM registry
Source: Clin Transl Oncol. 2024 Feb 15;26(7):1674–86. doi: 10.1007/s12094-024-03388-6 (PMC11178610; doi:10.1007/s12094-024-03388-6)
Supplement: Supplementary file 6 — Supplementary file6 (DOCX 16 KB) [file 12094_2024_3388_MOESM6_ESM.docx]

**Annex table 4.** Exploratory analysis of the interaction between chemotherapy regimen and statistically significant covariates of the COX model for PFS.

| **Covariates** | **HR** | **95% CI HR** |
| --- | --- | --- |
| **ECOG-PS**  *CP*  *FOLFOX # 0*  *FOLFOX # 1*  *FOLFOX # ≥2*  *CAPOX # 0*  *CAPOX # 1*  *CAPOX # ≥2*  *FP # 0*  *FP # 1*  *FP # ≥2* | Ref.  0.9286  0.6849  0.6725  1.0437  0.8140  0.7783  1.9502  0.6461  1.6578 | Ref.  0.5639 - 1.5290  0.5104 - 0.9191  0.4107 - 1.1012  0.6504 - 1.6748  0.6161 - 1.0753  0.4559 - 1.3288  0.9334 - 4.0746  0.4388 - 0.9512  0.4898 - 5.6110 |
| **Metastatic sites**  CP  *FOLFOX # < 2*  *FOLFOX # > 2*  *CAPOX # < 2*  *CAPOX # > 2*  *FP # < 2*  *FP # > 2* | Ref.  0.7972  0.7062  1.0002  0.7768  0.9144  0.7588 | Ref.  0.5495 - 1.1566  0.5320 - 0.9375  0.7008 - 1.4275  0.5863 - 1.0293  0.5375 - 1.5556  0.5013 - 1.1484 |
| **Bone metastases**  *CP*  *FOLFOX # No*  *FOLFOX # Yes*  *CAPOX # No*  *CAPOX # Yes*  *FP # No*  *FP # Yes* | Ref.  0.7357  0.6161  0.9089  0.4840  0.7901  1.1034 | Ref.  0.5755 - 0.9406  0.3131 - 1.2124  0.7216 - 1.1448  0.2297 - 1.0198  0.5614 - 1.1119  0.3453 - 3.5259 |
| ***Albumin***  *CP*  *FOLFOX # Normal*  *FOLFOX # < 35 g/dl*  *CAPOX # Normal*  *CAPOX # < 35 g/dl*  *FP # Normal*  *FP # < 35 g/dl* | Ref.  0.7882  0.6259  0.8403  0.9222  0.7737  0.8525 | Ref.  0.6085 - 1.0211  0.3875 - 1.0109  0.6601 - 1.0697  0.5532 - 1.5375  0.5262 - 1.1378  0.4482 - 1.6213 |
| **NLR**  *CP*  *FOLFOX*  *CAPOX*  *FP* | Ref.  0.6762  0.7844  0.7973 | Ref.  0.5029 - 0.9093  0.5731 - 1.0736  0.5336 - 1.1912 |

Abbreviations: ECOG-PS, Eastern Cooperative Oncology Group Performance Status; NLR, neutrophil-to-lymphocyte ratio; HR, hazard ratio; CI, confidence interval.
