# Supplementary material for: Is there a preferred platinum and fluoropyrimidine regimen for advanced HER2-negative esophagogastric adenocarcinoma? Insights from 1293 patients in AGAMENON–SEOM registry
Source: Clin Transl Oncol. 2024 Feb 15;26(7):1674–86. doi: 10.1007/s12094-024-03388-6 (PMC11178610; doi:10.1007/s12094-024-03388-6)
Supplement: Supplementary file 7 — Supplementary file7 (DOCX 15 KB) [file 12094_2024_3388_MOESM7_ESM.docx]

**Annex Table 5.** Exploratory analysis of the interaction between based-platinum regimen and statistically significant covariates of the COX model for SLP.

| **Covariates** | **HR** | **95% CI HR** |
| --- | --- | --- |
| **ECOG-PS**  Cisplatin  *Oxaliplatin # 0*  *Oxaliplatin # 1*  *Oxaliplatin # ≥2* | Ref.  0.8700  0.8848  0.6997 | Ref.  0.5890- 1.2759  0.7095 – 1.1034  0.4533 – 1.0801 |
| **Metastatic sites**  *Cisplatin*  *Oxaliplatin # < 2*  *Oxaliplatin # > 2* | Ref.  0.9361  0.7996 | Ref.  0.7051 – 1.2426  0.6374 – 1.0031 |
| **Bone metastases**  *Cisplatin*  *Oxaliplatin # No*  *Oxaliplatin # Yes* | Ref.  0.8849  0.5559 | Ref.  0.7339 – 1.0670  0.3138 - 0.9848 |
| ***Albumin***  *Cisplatin*  *Oxaliplatin # Normal*  *Oxaliplatin # < 35 g/dL* | Ref.  0.8734  0.7696 | Ref.  0.7159 - 1.0655  0.5322 - 1.1129 |
| **NLR**  *Cisplatin*  *Oxaliplatin* | Ref.  0.7800 | Ref.  0.6191 – 0.9828 |

Abbreviations: ECOG-PS, Eastern Cooperative Oncology Group Performance Status; NLR, neutrophil-to-lymphocyte ratio; HR, hazard ratio; CI, confidence interval.
